# Role of Mesenchymal Stem Cell Exosomes and Injectable Platelet Rich Fibrin on Structure and Function of Submandibular Salivary Gland of Aged Albino Rats

**DOI:** 10.1007/s44445-025-00056-5

**Published:** 2025-09-09

**Authors:** Hoda O. Desouky, Ahmed M. Halawa, Rabab Hassan

**Affiliations:** 1 Oral Biology Department, Faculty of Dentistry, Ain Shams University, Cairo, Egypt; 2 Oral Biology Department, Faculty of Dentistry, Misr International University, Cairo, Egypt

**Keywords:** Submandibular salivary gland, Stem cell exosomes, Injectable platelet rich fibrin, Aquaporin 5, Nerve growth factor

## Abstract

To compare the efficacy of using bone marrow mesenchymal stem cell (BM-MSC) exosomes and injectable platelet rich fibrin (i-PRF) on the submandibular salivary glands (SMGs) of aged albino rats in restoring salivary gland structure and function. A total of 40 healthy male albino rats were used, two for obtaining the BM-MSCs, 10 for i-PRF preparation and seven adult rats (6-8 months old) represented the control group (Group 1). The remaining 21 rats were aged (18-20 months old) and divided into three groups of seven rats each; (Group 2): received no treatment, (Group 3): each rat received a single intraglandular injection of BM-MSC exosomes (50 μg/kg/dose suspended in 0.2 ml PBS), and (Group 4): each rat received a single intraglandular injection of i-PRF (0.2 mL). One month later, glands were dissected and examined histologically for structural changes. Function was assessed via immunohistochemical examination using aquaporin-5 (AQP5) and enzyme linked immunosorbent assay (ELISA) for nerve growth factor (NGF) then analyzed statistically. Histologically, Group 1 showed normal acini and duct histology. Group 2 showed structural degeneration in acini and different duct systems. Treated groups represented signs of regeneration in the form of uniform duct systems and acini similar to Group 1. Immunohistochemical examination revealed increased immuno-expression of AQP5, while ELISA showed decreased NGF in all treated groups in relation to the aged group, and this was proven statistically. Aging causes deterioration in structure and function of the SMGs. BM-MSC exosomes and i-PRF can alleviate the damaging effect of aged SMGs.

## Introduction

The submandibular gland (SMG) is the second-largest salivary gland (SG) and secretes a saliva composed mainly of mucins, enzymes, inorganic substances, and signaling molecules essential for lubrication and protection of the oral mucosa (Xu et al. 2019). In addition, the SMG expresses aquaporin-5 (AQP5) protein, which plays a significant role in fluid secretion in SG tissue (Delporte et al. 2016), as well as nerve growth factor (NGF) which demonstrated an essential role in the repair of damaged cells in the oral mucosa (Chibly et al. 2022).

The function of the SMG is influenced by many factors which may include pathological conditions such as cysts and tumors or physiological factors such as aging (Toan & Ahn 2021).

Salivary gland aging is a multistage, multifactorial process which frequently leads to impairment of function (Li et al. 2023). This is due to several cellular and molecular mechanisms, including a decline in stem cell number and proliferative activity of SG stem cells. Another reason is the inefficiency of the cellular turnover system, causing senescent cells to accumulate in aged tissues and release reactive oxygen species (ROS), creating a harsh environment for other non-senescent cells (Gorgoulis et al. 2019). Aging not only causes cellular alterations but rather induces a persistent pro-inflammatory state in aged tissues known as “inflammaging,” which can increase inflammatory factors, lipid accumulation, fibrosis, and oxidative stress, all of which contribute to cellular senescence and diminished glandular function (Kim et al. 2023).

Aged SGs exhibit structural changes, alteration in salivary composition, and changes in salivary flow rate (Toan & Ahn 2021), which leads to diminishing oral and overall health (Nam et al. 2019).

Tissue engineering is one of the recent regenerative techniques which overcome age changes by using life sciences and materials engineering to produce tissue substitutes that mimic the structure and function of their natural counterparts within the body. This technique involves using stem cells, biomaterials, and biochemical signals to recreate the natural organ environment (Kwon et al. 2018).

Despite its importance, the use of stem cells does have limitations, including the risk of mutagenic tumorigenesis, potential contamination, immune rejection, and ethical dilemmas (Zou et al. 2016). Consequently, there is currently a growing focus on finding ways to avoid the shortcomings of these limitations while maximizing the benefits of stem cell therapy (Mousaei et al. 2022).

Exosomes are lipid bilayer-enclosed, extracellular, nano-sized vesicles (30-200 nm) released by most cells and are thought to play key roles in intercellular communication via the transfer of genetic molecules such as coding and non-coding RNAs (Willms et al. 2018). They have been found to mediate the paracrine action of mesenchymal stem cells (MSCs) (Xie et al. 2016). Hence, they play a role in cell-free therapy for treating many diseases (Muralikumar et al. 2021).

Platelet rich concentrates have also been widely used in the medical and dental field for their regenerative properties. Platelet rich fibrin (PRF) is a second-generation modification of platelet rich plasma (PRP), formulated without an anticoagulant to overcome the drawbacks of first-generation PRP, promoting more natural and faster tissue regeneration. A few years ago, it was developed in an injectable form, injectable PRF (i-PRF), for a longer and more sustained release of growth factors (Varela et al. 2019).

From the previous information, this study prospectively evaluated the efficacy of using BM-MSC exosomes and i-PRF as a therapy for the SMGs of aged albino rats in restoring SG structure and function.

## Materials and methods

This experiment was conducted in accordance with the ARRIVE 2.0 (Animal Research: Reporting of In Vivo Experiments) guidelines to ensure ethical animal research and in accordance with the research guidelines of the Research Ethics Committee, Faculty of Dentistry, Ain Shams University (FDASU-Rec IR/D022223).

The animals were anesthetized during all painful or discomfort procedures. The researchers were kind to the animals and were aware of and followed the Five Freedoms that outline the aspects of animal welfare under human control: 1. Freedom from hunger or thirst, 2. Freedom from discomfort, 3. Freedom from pain, 4. Freedom to express normal behavior, 5. Freedom from fear and distress. Additionally, Three Rs (3Rs): 1. Replacement: non-animal computer models could not be used to achieve the scientific aims of the research, 2. Reduction: the least possible number of animals was used that can provide sufficient statistical power, 3. Refinement: all means to alleviate or minimize potential pain, suffering or distress of the animals were performed.

Sample size calculation was performed using G*Power version 3.1.9.7 based on the results of a previous study (Cui et al. 2021). A power analysis was designed to have adequate power to apply a two-sided statistical test to reject the null hypothesis that there is no difference between groups. By adopting an alpha level of (0.05) and a beta of (0.1), i.e. power = 90% and an effect size (d) of (0.8265) calculated based on the results of (Cui et al. 2021). The predicted sample size (n) was (28), i.e., 7 rats per group.

### Isolation and culture of BM-MSCs

The BM-MSCs were attained from two, ten-week-old male albino rats at the Biochemistry and Microbiology Unit, Faculty of Medicine, Cairo University.

From the tibia, bone marrow was flushed using Phosphate Buffered Saline (PBS) after which it was centrifuged at 1000 rpm for 5 min. After discarding the upper layer, a mononuclear cell layer remained, then washed twice using PBS and centrifuged at 200 × g for 10 min at 10 °C. Culturing of isolated BM-MSCs using 10% fetal bovine serum (GIBCO™/Bethesda Research Laboratories, USA) was performed, after which cells were incubated at 37 °C in a 5% humidified CO_2_ incubator (Innova® CO-170, UK) until 80–90% confluence was reached (Zuo et al. 2019).

### Isolation and identification of exosomes

The supernatants of the third passage of BM-MSCs (5 × 10^6^ cells/ml) were collected to obtain the exosomes and then 0.5% bovine serum albumin (Sigma® Chemical Co., USA) was added as supplementation. The cells were centrifuged at 2000 × g for 20 min to remove any debris. The cell-free supernatants were ultracentrifuged at 100,000 × g using an ultracentrifuge (Beckman Coulter®, Optima L 90 K, USA) at 4 °C for 1 hr. Next, the supernatants were washed in serum-free medium 199 containing 25 mmol of 4-(2-hydroxyl ethyl)−1-piperazine ethane sulfonic acid (Sigma® Chemical Co., USA), followed by ultracentrifugation for a second time to obtain the purified exosomes that were cultured overnight in exosome collection medium (Yan et al. 2017). Their identification was established using transmission electron microscopy (HITACHI, H-7650, Japan).

### Injectable Platelet Rich Fibrin (i-PRF) Preparation

Preparation of i-PRF was performed by obtaining blood from the retro-orbital plexus of 10 male albino rats. Whole blood was collected into sterile tubes and centrifuged at 800 RPM for 3 min (60x g) at room temperature by a Duo Centrifuge (Thermo Fisher Scientific, USA), resulting in separation of the red blood cells (RBCs) and plasma (containing platelets and leukocytes) (Liang et al. 2021). Following centrifugation, the blood cells precipitated at the bottom, and the yellow tinted plasma was collected from the upper half.

### Animals

Twenty-eight, healthy, male Wistar albino rats were used, seven rats were 6-8 months old (200–250 gm) and 21 rats were 18-20 months old (300–350 gm). The rats were placed in metal cages (3-4 rats/cage) at the Medical Research Center, Faculty of Medicine, Ain Shams University and were supervised by a specialized veterinarian. The rats were given access to water and food ad libitum throughout the experimentation period.

### Animal Grouping

Following one week of acclimatization, rats of 6-8 months old served as the adult control group, while the remaining 21 rats of 18-20 months old were randomly distributed into three groups, seven rats each, so the animal grouping was as follows:

- Group 1 (Adult): consisted of adult rats (6-8 months old) and did not receive any intervention.
- Group 2 (Aged): consisted of aged rats (18-20 months old) and did not receive any intervention.
- Group 3 (Aged treated with BM-MSC Exosomes): consisted of aged rats that received a single intraglandular injection of exosomes (50 μg/kg/dose suspended in 0.2 ml PBS injected into the submandibular gland as 0.1 mL per lobe) (Zhang et al. 2020; Mahmoud et al. 2023).
- Group 4 (Aged treated with i-PRF): consisted of aged rats that received a single intraglandular injection of i-PRF (0.2 ml injected into the submandibular gland as 0.1 mL per lobe) (Erdur et al. 2021; Mahmoud et al. 2023).

### Application of treatment

The intraglandular injections were performed using the closed surgical technique. Treatments were loaded in an insulin syringe and slowly injected into the SMG under indirect vision.

### Specimens collection and sample preparation

After one month of treatment, rats were euthanized using overdose of ketamine. Carcasses and wastes of the used animals were hygienically disposed of by incineration in the Medical Research Center (Faculty of Medicine, Ain Shams University). The right and left lobes of the SMGs were excised. The right lobes of the SMGs were washed in saline solution and fixed in 10% buffered formalin for 48 hr for histological staining using hematoxylin & eosin (H&E) and immunohistochemical staining using AQP5. The left lobes of the SMGs were kept in −20°C in Eppendorf tubes and processed for enzyme linked immunosorbent assay (ELISA).

Blinding technique was performed by coding the collected samples from all study groups, so the authors remained unaware of the group assignment during examination, assessment and analysis phases.

#### H&E Staining

The SMG tissue was dehydrated in ascending grades of ethyl alcohol, cleared in xylene and embedded in paraffin. Sections of 4-5 µm in thickness were obtained using a Leica RM2245 microtome and collected on microscope slides. Tissue sections were deparaffinized and rehydrated with descending grades of ethyl alcohol then stained with H&E for histological investigation (Dunn et al. 2024).

#### Immunohistochemical staining

The sections of SMG tissue were mounted on positively charged microscope slides. Sections were deparaffinized and submitted to antigen recovery using sodium citrate buffer pH (6.0) for 15 min in a microwave. The sections were then washed in distilled water and subjected to endogenous peroxidase blocking for 15 min in the dark and to unspecific protein blocking at 3% in PBS for 1 hr. Sections were then incubated with AQP5 antibodies (catalog No. E-AB-15511, Elabscience®, USA) at concentration 1:50 overnight at 4°C. Next, sections were washed in PBS and incubated with secondary antibodies at concentration 1:100 for 2 hr at room temperature. The slides were revealed using DAB chromogen and counterstained with hematoxylin for 1 min (Araujo et al. 2018). Expression of AQP5 appeared as a brown cytoplasmic stain localized in the striated and granular convoluted ducts (Cui et al. 2021).

The yielded slides were examined and photographed using a digital camera (C5060 Olympus, Japan) mounted on a light microscope (BX60, Olympus, Japan) at magnification x200 and x400.

#### Preparation for ELISA

The SMGs were rinsed thoroughly in ice-cold PBS (pH 7.4) to remove excess blood and weighed then homogenized in PBS with a glass homogenizer then centrifuged for 5 min at 5000xg to retrieve the supernatant. Standard solution was prepared by adding 120μl of the standard (3200ng/L) to 120μl of standard diluent to generate a 1600ng/L standard stock solution and left for 15 min with gentle agitation prior to making two-fold serial dilutions. Standard diluent served as the zero standard (0 ng/ml). 50μl standard was added to the standard well, while 40 μl of the sample were added to the sample wells and then 10 μl NGF (Catalog No. E0539Ra, Bioassay Technology Lab, USA) was added. Streptavidin-HRP was then added to sample wells and standard wells. They were mixed well then incubated for 60 min at 37°C. After washing, 50 μl of substrate solution A followed by 50 μl of substrate solution B were added to each well. The plate was incubated for 10 min in the dark at 37°C. Then 50 μl of stop solution was added to each well, after which the blue color changed immediately to a yellow color. The optical density of each well was determined using a microplate reader (Multiskan™ SkyHigh, Life Technologies, Thermo Fisher Scientific, Singapore) at 450 nm (Britt et al. 2022). The entire ELISA procedure was performed in the Central Lab of Stem Cells and Biomaterials Applied Research (CLSCBAR).

### Histomorphometric Analysis

Five representative fields per section stained with anti-AQP5 antibody were captured at x400 magnification. The immune positive area fraction (area%) was measured using Image J software (Version 1.41a, NIH, USA). Images were first manually corrected for brightness and contrast and then converted into 8-bit type gray scale. Color thresholding was then performed and calculated automatically.

### Statistical Analysis

Numerical data from histomorphometric analysis and ELISA were analyzed statistically by using SPSS 23.0 (SPSS, Inc., Chicago, IL, USA) and presented as mean, standard deviation and confidence intervals. Data were explored for normality by checking the data distribution using Kolmogorov-Smirnov and Shapiro-Wilk tests. Comparisons between different studied groups were performed by one way analysis of variance (ANOVA) test, followed by Bonferroni’s post hoc test for pairwise comparison. *P*-value was highly significant, significant or non-significant at ≤0.001, ≤0.05 or > 0.05 respectively.

## Results

### Characterization of BM-MSCs Exosomes

**Fig. 1:**
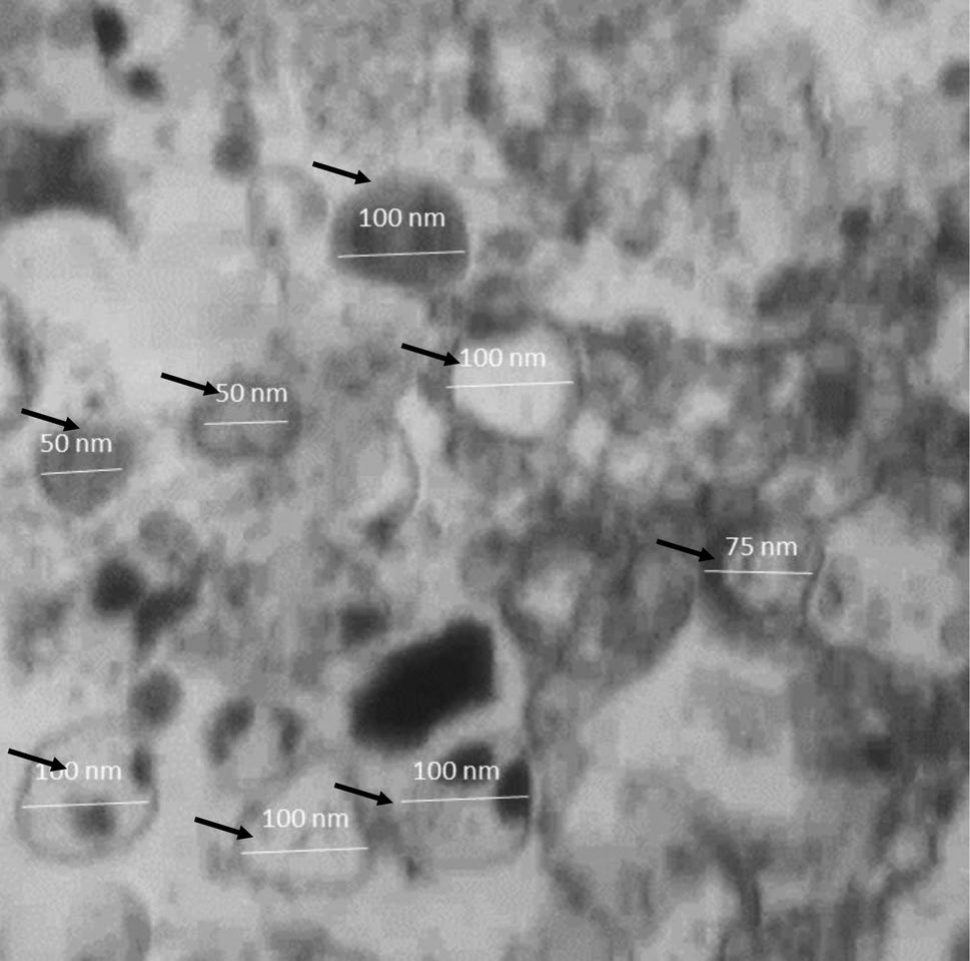
Transmission electron micrograph showing the distinctive morphology of the double-membrane-bound spheroids (arrows) of the BM-MSCs exosomes; their diameters ranging from 50 to 100 nm

Mild to moderate electron lucent, double-membrane-bound, spheroid-shaped exosomes with 50-100 nm in diameter were observed (Fig. 1).

### Histological results

#### Acini, Granular Convoluted Tubules and Striated Ducts

**Fig. 2:**
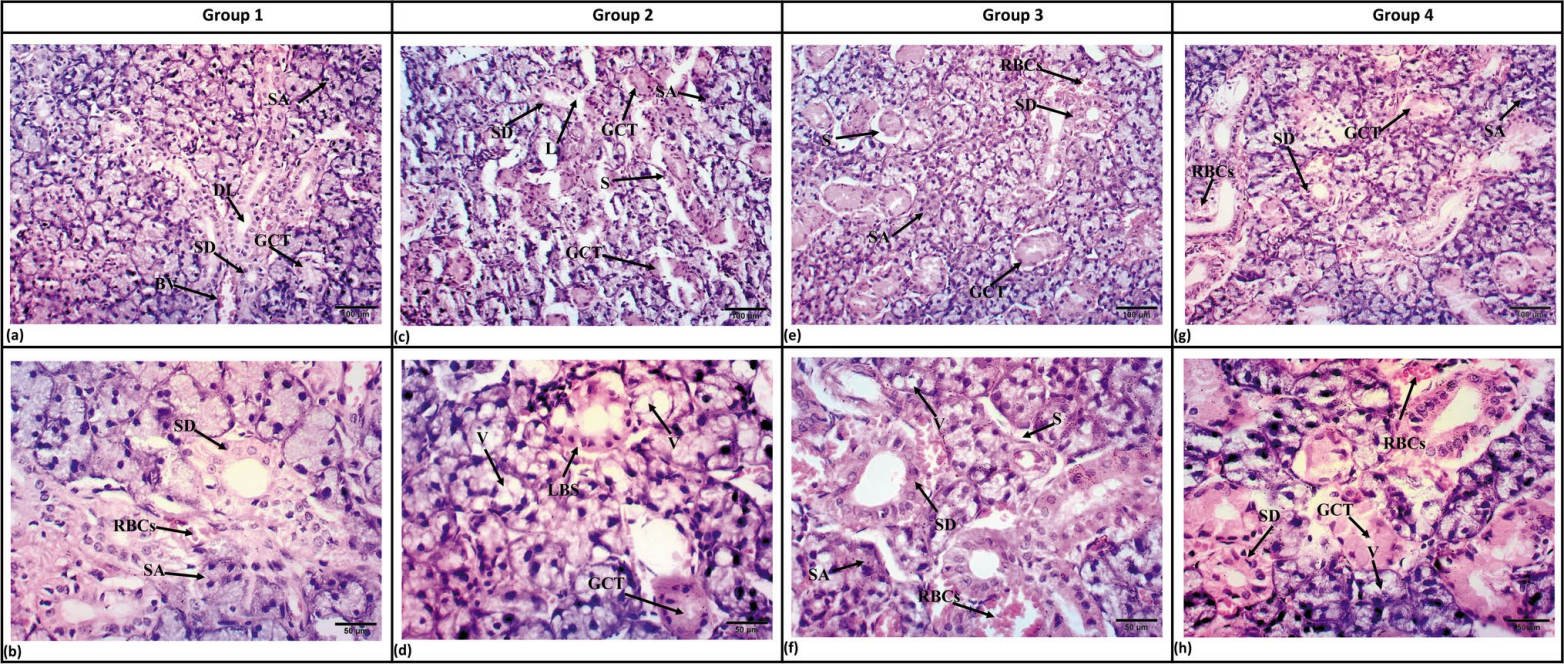
Photomicrographs of SMG showing: Group 1 (**a**) Serous acini, striated ducts with empty duct lumen, and glomerular convoluted tubules, blood vessels filled with RBCs. (**b**) Spherical serous acini made of pyramidal cells and basal nuclei. Striated ducts with columnar cells, central nuclei and eosinophilic basal striations. Blood vessels filled with RBCs. Group 2 (**c**)- Spacing between the acini, degenerated GCTs with signs of degeneration and vacuolization, striated ducts with loss of duct continuity in some areas. (**d**) Serous acini intracytoplasmic vacuolization, GCTs vacuolization, loss of basal striations in striated ducts. Group 3 (**e**)- Spherical, less spaced serous acini, less atrophied GCTs, regular striated ducts, and extravasated RBCs between acini. (**f**) Some spacing between the acini with intracytoplasmic acinar vacuolization, and blood vessels filled with RBCs. Group 4 (**g**) Closely packed serous acini, GCTs, striated ducts, and extravasated RBCS. (**h**) Some vacuolization of acinar, striated ducts with basal striations and extravasated RBCs. SA, Serous acini; SD, Striated ducts; GCT, Granular convoluted tubules; DL, Duct lumen; S, Spacing between acini; V, Vacuolization L, Loss of duct continuity; LBS, Loss of basal striations; BV, Blood vessel; RBCs, Red blood cells. (H&E, original magnification a, c, e & g x200, scale bar 100 µm, b, d, f, & h x400, scale bar 50 µm)

Histological examination of SMGs of Group 1 revealed normal architecture. Serous acini were closely packed and spherical shaped with a narrow lumen. They showed cells of pyramidal shape with basal nuclei and a basophilic cytoplasm. Granular convoluted tubules (GCTs) showed epithelial cells which were columnar in shape with basal nuclei in an eosinophilic cytoplasm. Striated ducts of this group showed columnar cells with central nuclei situated in an eosinophilic cytoplasm surrounding a wide and empty lumen. Inter-acinar blood vessels filled with RBCs were noticed (Fig. 2a & b).

Group 2 showed widely spaced and degenerated acini as well as acinar cells with cytoplasmic vacuolation. The GCTs appeared atrophied. Striated ducts showed thinning in the lining of the duct and loss of duct continuity and basal striation, in addition to cytoplasmic vacuolization in some cells (Fig. 2c & d).

Both Group 3 (Fig. 2e & f) and Group 4 (Fig. 2g & h) showed less spaced spherical acini with regular nuclei and intracytoplasmic vacuoles. Apparent reduction in GCTs atrophy and intracytoplasmic vacuoles were detected. Striated ducts showed almost regular histological picture. The treated groups also showed dilated blood vessels with extravasated RBCs.

#### Excretory ducts

**Fig. 3:**
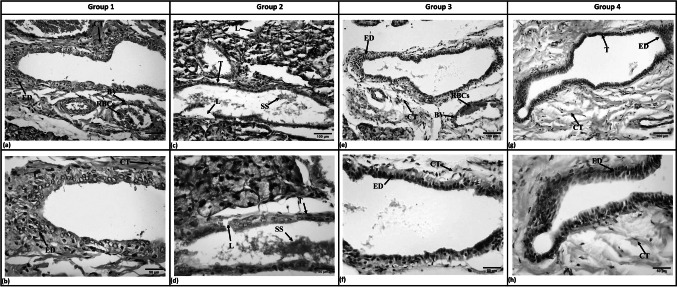
Photomicrographs of SMG excretory ducts showing: Group 1 (**a**) Wide, empty duct lumen and fibrous connective tissue and blood vessels filled with RBCs. (**b**) Pseudostratified columnar epithelium lying in organized, fibrous connective tissue. Group 2 (**c**) Loss of pseudostratification of lining cells, thinning of duct lining, loss of duct continuity and stagnant secretions. (**d**) Thinning of duct lining, loss of continuation, and stagnated secretions. Group 3 (**e**) Regular duct with wide lumen and dilated blood vessels engorged with RBCs. (**f**) Pseudostratified epithelium surrounded with fibrous connective tissue. Group 4 (**g**) Regular duct thickness surrounded by organized connective tissue. (**h**) Pseudostratified epithelium and fibrous connective tissue. (ED, Excretory duct; CT, Connective tissue; BV, Blood vessels; RBCs, Red blood cells; SS, Stagnant secretion; T, Thinning of duct lining; L, Loss of duct continuity (H&E, original magnification a, c, e & g x200, scale bar 100 µm, b, d, f, & h x400, scale bar 50 µm)

In Group 1, the excretory ducts showed a wide and empty lumen which was lined by pseudostratified columnar epithelial cells along with goblet cells. The ducts were surrounded by organized connective tissue fibers with blood vessels (Fig. 3a & b).

Excretory ducts of Group 2 showed thinning of the epithelial linings in some parts with loss of epithelial continuity and pseudostratification in other parts. The lumens showed stagnated secretions, while the surrounding connective tissue showed scanty collagen fibers (Fig. 3c & d).

In both treated groups, the excretory ducts showed regular thickness of the epithelial lining and wide lumen with some stagnant secretions (Fig. 3e, f, g & h). The surrounding fibrous connective tissue was infiltrated with blood vessels of varying size and thickness filled with RBCs in Group 3 (Fig. 3e). Meanwhile, it appeared organized in Group 4 (Fig. 3g).

### Immunohistochemical and Statistical Results

**Fig. 4:**
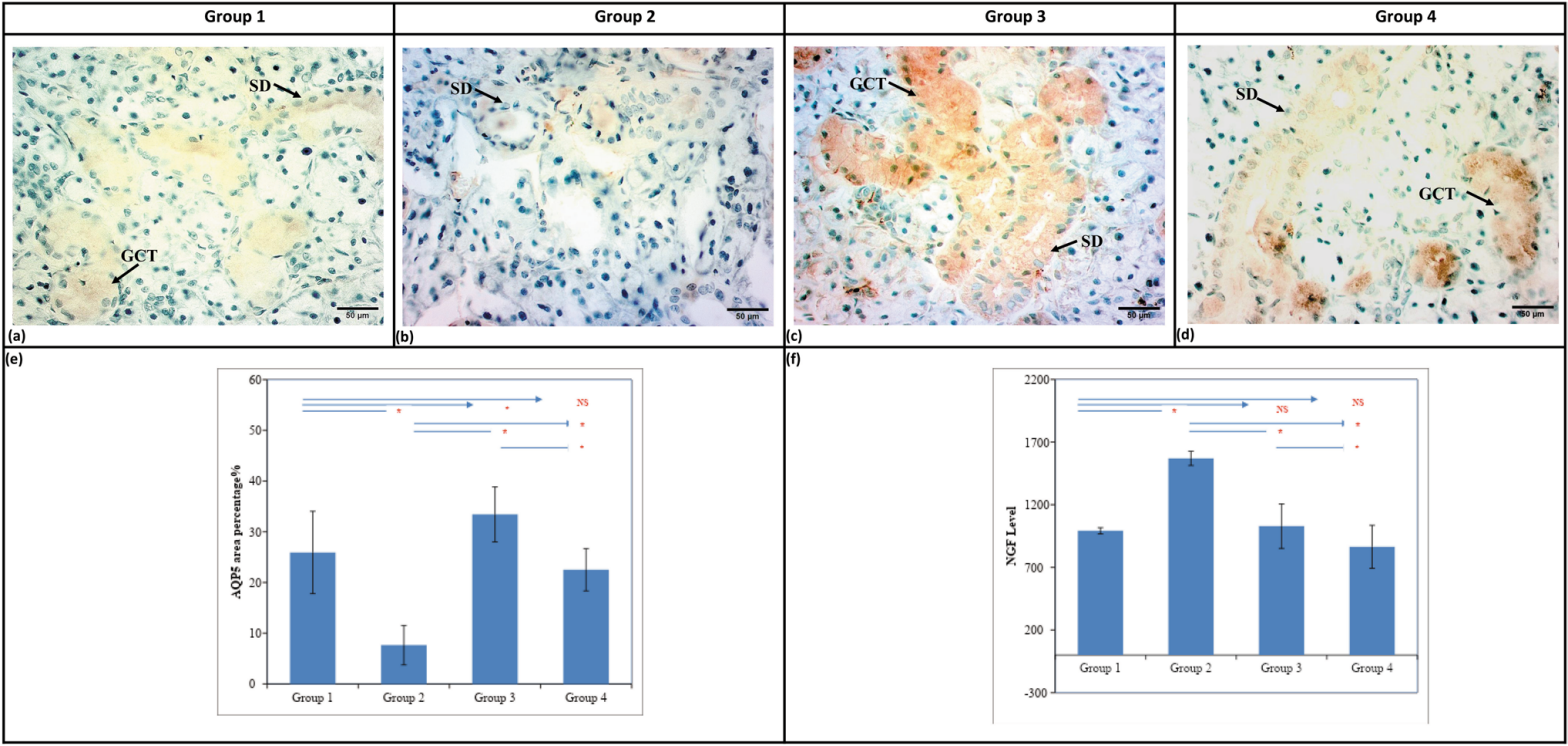
Photomicrographs of SMG showing positive cytoplasmic immunoreaction in striated duct and GCT cells in (**a**) Group 1, (**b**) Group 2, (**c**) Group 3, (**d**) group 4 (Anti-AQP5 antibody, original magnification x400, scale bar 50 µm). Bar charts representing mean and SD values of: (**e**)- AQP5 area% and (**f**)- NGF level of the different groups. (* Significant; ** Highly significant; NS: non-significant)

Positive immuno-expressions were located in the GCTs and striated ducts of all groups (Fig. 4a, b, c & d). Statistically high significant difference was recorded between all groups. Group 3 showed the highest mean area% of positive immuno-expressions, followed by Group 1, then Group 4, which showed no statistically significant difference to Group 1. Group 2 showed the least mean area% (Table 1, Fig. 4e).

**Table 1:** Showing the mean ± SD values, results of ANOVA and post hoc tests for the comparison between different groups regarding AQP5 area percentage

Groups	Mean	±SD	±SE	95% C.I. for Mean		Min.	Max.	F-test	*p-*value
				Lower	Upper				
Group 1 (Adult)	25.92B	8.12	2.45	20.46	31.38	12.56	40.74	28.984	0.001*
Group 2 (Aged)	7.64C	3.89	1.38	4.39	10.90	1.68	14.38		
Group 3 (Aged treated with BM-MSC Exosomes)	33.42A	5.42	1.81	29.26	37.59	25.70	42.40		
Group 4 (Aged treated with i-PRF)	22.51B	4.17	1.32	19.53	25.49	14.92	28.88		

*SD* Standard deviation, *SE* Standard Error *Min.* Minimum, *Max.* Maximum, *C.I.* Confidence Interval Significance level *p*≤0.05, *significant; Bonferroni’s post hoc: Means sharing the same superscript letter are not significantly different

### NGF Level and Statistical Results

The highest mean value was recorded in Group 2 followed by Group 3 and then Group 1. The least value recorded was in Group 4. ANOVA test revealed a statistically significant difference between groups (*P*=0.001). Bonferroni’s post hoc test revealed no significant difference between group 1, 3 and group 4 (Table 2, Fig. 4f).

**Table 2:** Showing the mean ± SD values, results of ANOVA and post hoc tests for the comparison between different groups regarding NGF level

Groups	Mean	±SD	±SE	95% C.I. for Mean		Min.	Max.	F-test	*p*-value
				Lower	Upper				
Group 1 (Adult)	992.0B	24.9	11.1	961.1	1022.9	950	1010	27.668	0.001*
Group 2 (Aged)	1570.0A	57.0	25.5	1499.2	1640.8	1500	1650		
Group 3 (Aged treated with MSC Exosomes)	1028.6B	177.6	67.1	864.3	1192.8	900	1400		
Group 4 (Aged treated with i-PRF)	864.3B	171.6	64.8	705.6	1022.9	700	1090		

*SD* Standard deviation, *SE* Standard Error, *Min.* Minimum, *Max.* Maximum, *C.I.* Confidence Interval Significance level *p*≤0.05, *significant; Bonferroni’s post hoc: Means sharing the same superscript letter are not significantly different

## Discussion

Submandibular glands provide most of salivary secretions during resting condition (Ekström et al. 2017) and reveal the most notable structural degeneration with aging​ (Toan & Ahn 2021). The regenerative potential of BM-MSC exosomes and i-PRF improve the quality of life of geriatric patients by reversing the symptoms associated with the age changes of SGs (Sawan et al. 2021).

The BM-MSC exosomes and i-PRF in the present study were selected because they contain a majority of bioactive substances and high concentrations of growth factors that easily bypass all biological barriers to reach target tissues while limiting side effects (Miron et al. 2017; Sun et al. 2022).

In the current study, ultracentrifugation was used as it is one of the simplest methods for exosome isolation and is based on molecular size (Li et al. 2017).

In this study, the intraglandular injection of BM-MSC exosomes and i-PRF was the route of choice as it allows for less dosage to be injected and is less time consuming (Turksen 2023).

In this study, AQP5 was the choice marker given its importance in both maintaining the normal physiology of the gland as well as regeneration of SGs (Hosoi et al. 2020). Meanwhile, there are two types of NGF, pro-NGF (the major form of NGF in saliva), which is the unprocessed precursor form that is cleaved intercellularly to give the second type, which is mature NGF (Schenck et al. 2017; Liu et al 2021). Hence the pro-NGF level was evaluated in this study as it plays a regulatory role in SG cell growth and differentiation (Li et al. 2021).

In the present work, the structural histological changes reported in Group 2 were in agreement with (Elsaied 2019). This could be attributed to the increased ROS production and oxidative stress environment characteristic of the aging process (Ogrodnik et al. 2019) and the decline in stem cell number and proliferative activity of stem cells (Takamatsu et al. 2021). The cellular cytoplasmic vacuolization might be related to the edematous changes and fatty degeneration associated with aging as well as the decreased secretory function of the acini (Elghonamy et al. 2018; Elsaied 2019). Moreover, the acinar atrophy and spacing could result from a decrease in the intercellular adhesion caused by the reduction in the expression of E-cadherin (Li et al. 2023).

In this study, the degenerative changes reported in the duct system in Group 2 are in agreement with (Toan & Ahn 2021; Rabea et al. 2022). The thinning of the ductal epithelium could be related to the downregulation of cytokeratin 7, a reliable marker of ductal epithelium. In addition, the stagnant secretions may be attributed to senescence, which causes reduction in mitochondria count and depletion of ATP (Mohamed et al. 2022), while the reported connective tissue changes could be linked to the increased rate of collagen fiber destruction due to the inflammatory environment associated with aging (Molière et al. 2023).

The histological results of Group 3 in the current study were in agreement with (AbuBakr et al. 2020). The recorded improvement may be linked to the ability of MSC-exosomes to promote intrinsic tissue regeneration by stimulating the proliferation of MSCs and their differentiation (Hong et al. 2018). Furthermore, it may be result of the antioxidant action of MSC-exosomes in alleviating tissue damage by reducing oxidative stress via neutralization of the ROS (Chen et al. 2023), their anti-inflammatory action by inhibiting the differentiation of pro-inflammatory T-cells (Qiao et al. 2023), and their microRNA 126 content which exerts anti-inflammatory action by reducing inflammatory cytokines (Ferguson et al. 2018; Nouralishahi et al. 2023).

The improvement in connective tissue and detectable presence of multiple blood vessels in Group 3 can be explained by the ability of exosomes to down-regulate matrix degrading enzymes such as matrix metalloproteinases (MMPs), up-regulate angiogenesis-related genes, and their preventive properties against the spread of inflammation associated with aging cells (Chen et al. 2018; Zhang et al. 2020; Xia et al. 2024).

In this study, the regenerative histological results recorded in Group 4 were in parallel with Elsherbini & Ezzat, (2020) who found that i-PRF increased the regenerative capacity in diabetic rats following a surgical defect in their SMGs by reducing caspase-3 and by increasing both angiogenesis and vascular endothelial growth factor. Furthermore, the reported improvement could be attributed to the ability of i-PRF to recruit stem cells from the blood stream for tissue regeneration (Melek and Said 2017), in addition to its anti-inflammatory action via reducing proinflammatory M1 phenotype of macrophages and inhibiting toll-like receptor 4, an activator of inflammatory stimulation (Zhang et al. 2020). The organized fibrous connective tissue reported in this group can be explained by Wang et al. (2017), who found that i-PRF promoted collagen synthesis and the release of pro-wound healing growth factors, platelet-derived growth factor and transforming growth factor-β.

In the current work, results of both immunohistochemistry and statistical analysis reported a reduction in AQP5-immunoreactivity area percentage of Group 2 in comparison to the control group, and these findings are in agreement with Bhattarai et al. (2018) who reported decreased responsiveness to AQP5 in aged rat SMGs. These results could be connected to abnormal expression, localization and/or trafficking of AQP5 within the gland (Delporte et al. 2016) and the increased oxidative stress and inflammatory response during aging (Liguori et al. 2018). Therefore, glandular destruction and disturbance in AQP5 expression induced by inflammation are responsible for decreased SG secretion and function. The highest AQP5 positive area% in Group 3 is in agreement with (Hu et al. 2023), who found that treatment with dental pulp stem cell exosomes up-regulated AQP5 expression and increased salivary flow rate in Sjogren’s Syndrome-related inflammation, while the high AQP5 positive area% in Group 4 could be related to the modulatory effect of i-PRF against the inflammatory process (Aydinyurt et al. 2021).

The ELISA and statistical analysis results of Group 2 showed increased concentration of NGF in comparison to the other groups. Our results are in parallel with Chen et al. (2024) who reported an increase in pro-NGF in aged rat lungs as it binds to the p75 neurotrophin receptor, which increases during aging. Fahnestock & Shekari, (2019) found that pro-NGF levels were increased in the cortex and hippocampus regions of the brain in Alzheimer's disease due to cognitive impairment and neurodegeneration associated with this illness. Moreover, Numakawa & Odaka, (2022) reported increased levels of pro-NGF in aged rodents due to impaired cleavage of pro-NGF by proteases such as MMPs and plasmin. The increase in pro-NGF levels in aged rats could be linked to the inflammatory condition which accompanies the aging process (Saavedra et al. 2023). Meanwhile, the concentration of pro-NGF in the treated groups was comparable to Group 1. Minnone et al. (2017) found that the p75 neurotrophin receptor to which pro-NGF binds is expressed on immune cells and regulated in the immune system, indicating that the role of NGF in the tissues depends on the state and activity of the immune cells and can directly affect the release of cytokines and inflammatory mediators from immune cells. Depending on NGF levels, the role of NGF in the tissue can be either pro-inflammatory or anti-inflammatory. This means that the effect of NGF on the tissues varies, and pro-NGF levels may both activate and decrease inflammation when needed (Ferraguti et al. 2024).

In the present study, treatment using BM-MSC exosomes statistically proved to be the most effective in restoring the structure and function of aged SMG, by showing the highest expression of both AQP5 and NGF. The possible explanation for our findings could be related to the paracrine effect of BM-MSC exosomes through carrying bioactive molecules including growth factors, chemokines, and cytokines, which directly interact with cells and influence tissue regeneration and repair (Ma et al. 2020), while i-PRF exhibits a generalized and passive release of growth factors in a slow and constant rate (Soyfoo et al. 2018).

Further investigations are needed to determine if better results can be achieved by altering the treatment dose or by combining both treatments.

## Conclusion

Aging results in deterioration in SMG architecture. BM-MSC exosomes and i-PRF are efficient in preserving morphology and minimizing the effects of aging on the SMG. Statistically, BM-MSC exosomes showed more improvement than i-PRF in AQP5 expression.

## Data Availability

The supporting data for the results of this study can be made available upon request.
